# Descriptions of a common belief in an 1813 Japanese beauty handbook regarding the influence of striped clothing on perceived body shape

**DOI:** 10.1177/20416695221130779

**Published:** 2022-10-12

**Authors:** Yuki Miyazaki, Kentaro Ishibashi

**Affiliations:** Department of Psychology, 12777 Fukuyama University, Japan; Hiroshima Prefectural Museum of History, Japan; Graduate School of Integrated Arts and Sciences, Hiroshima University, Japan

**Keywords:** striped clothes, Helmholtz illusion, fashion, body shape

## Abstract

“Clothes with horizontal (or vertical) stripes are perceived as wider and shorter (slimmer and taller).” This belief is common yet inconsistent with the Helmholtz illusion. It has often attracted attention from researchers of perception. Despite the controversy among empirical studies, it is persistently supported by the general public. This article explores the early appearance of this common belief in Japan in historical records. Consequently, we discovered the descriptions of the common belief in a Japanese beauty handbook titled “*Miyako Fuzoku Kewai Den* [Cosmetic manners and customs in Edo],” published in 1813. In Japan, this belief was not born in modern times. Instead, it was established over 200 years ago, when vertical striped patterns on clothes were popularized.

People wearing clothes with horizontal or vertical stripes are believed to be perceived as wider and shorter, or slimmer and taller, respectively. This common belief has attracted attention from researchers of perception because it is inconsistent with the Helmholtz illusion, where a square of the same area appears to be wider (or slimmer) in the case of a vertical (or horizontal) striped pattern (Helmholtz, 1867/1925, p. 193). Some studies have reported that, contrary to the common belief (i.e., consistent with the Helmholtz illusion), our body is perceived as slimmer by wearing clothes with horizontal (vs. vertical or no) stripes (Koutsoumpis et al., 2021; Thompson & Mikellidou, 2011). However, the effect of striped clothing depends on the body size of the wearer and the contexts (e.g., effects of presentation order of horizontal and vertical stripes) and varies widely across observers (Ashida et al., 2013). Counterexamples supporting the common belief have also been reported (Imai, 1982, see Ashida et al., 2013; Swami & Harris, 2012; Watham, 2012; Taya & Miura, 2007). Despite the controversy, the general public persistently supports this common belief about striped clothing and body shape.

When did this belief originate? This article explores the belief's early appearance in the historical record. Striped patterns (primarily vertical stripes) on clothes became popular in Japan’s late Edo period (approx. 1750–1868). Although stripes were used on clothes before this period (horizontal striped clothes were ubiquitous), the vertical striped pattern was considered rather undesirable for clothes (Maruyama, 2007). We explored materials describing beauty dating back to the late Edo period, when the patterns of vertical and horizontal stripes for clothes began to be popular.

We discovered descriptions of this common belief in a Japanese beauty handbook published in 1813 (Sayama & Hayami, 1813/1982). The handbook is titled “*Miyako Fuzoku Kewai Den* [Cosmetic manners and customs in Edo]” and was written by Hanshichimaru Sayama, a beautician. Shungyosai Hayami, a painter, illustrated it. This handbook is a comprehensive beauty guide, consisting of seven chapters in a three-volume set, describing makeup, skincare, hairstyles, how to dress, and so on. For example, the common belief about stripes is described on pages 9–12 of Chapter 6, Volume 3 (Figures 1 and 2).

**Figure 1.**
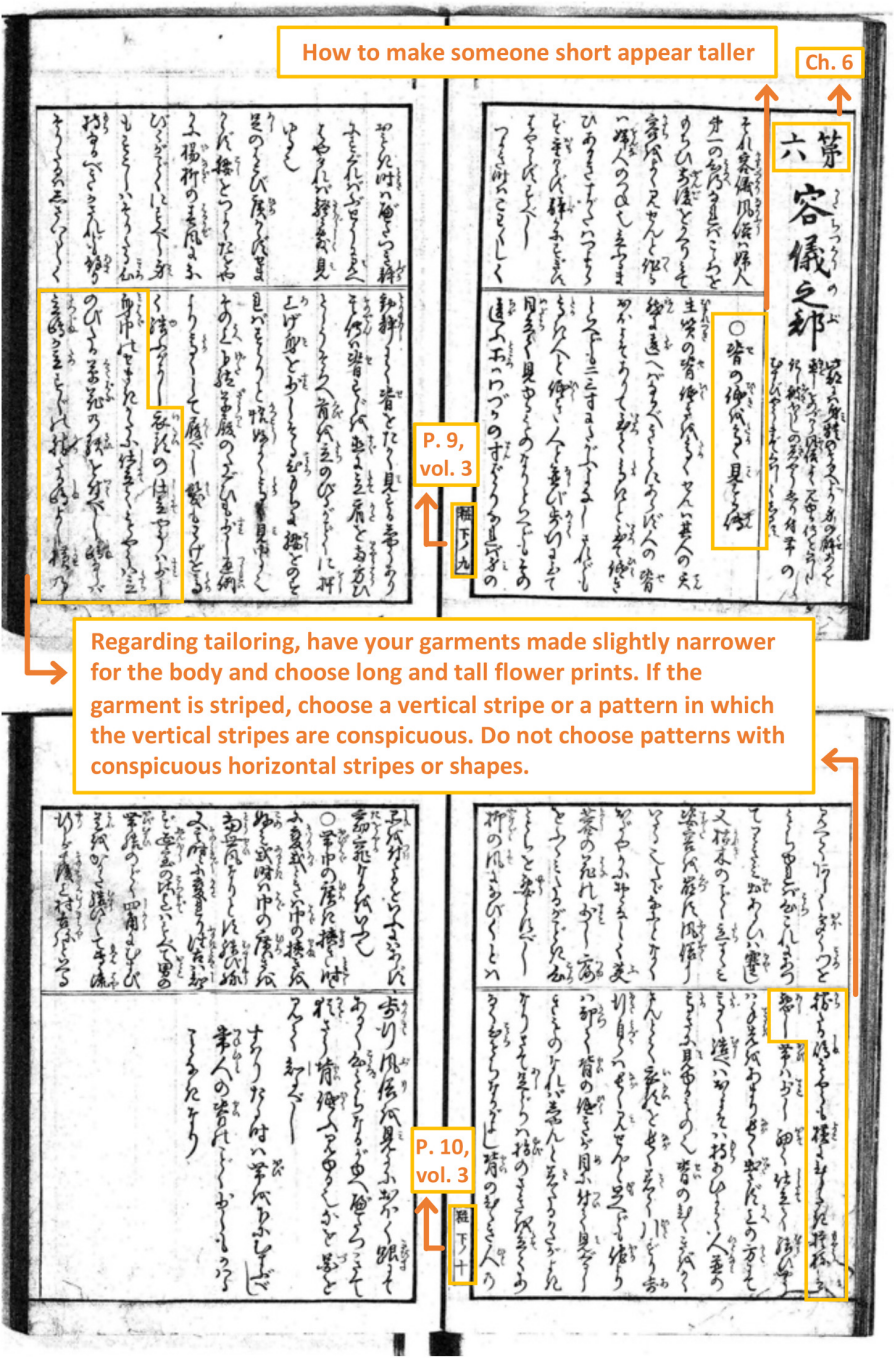
Pages 9–10, volume 3, “*Miyako Fuzoku Kewai Den*.”

*Note*. Created by modifying “*Miyako Fuzoku Kewai Den* (http://kotenseki.nijl.ac.jp/biblio/200005385).” The National Institute of Japanese Literature. Creative Commons Attribution License (CC BY-SA 4.0).

**Figure 2.**
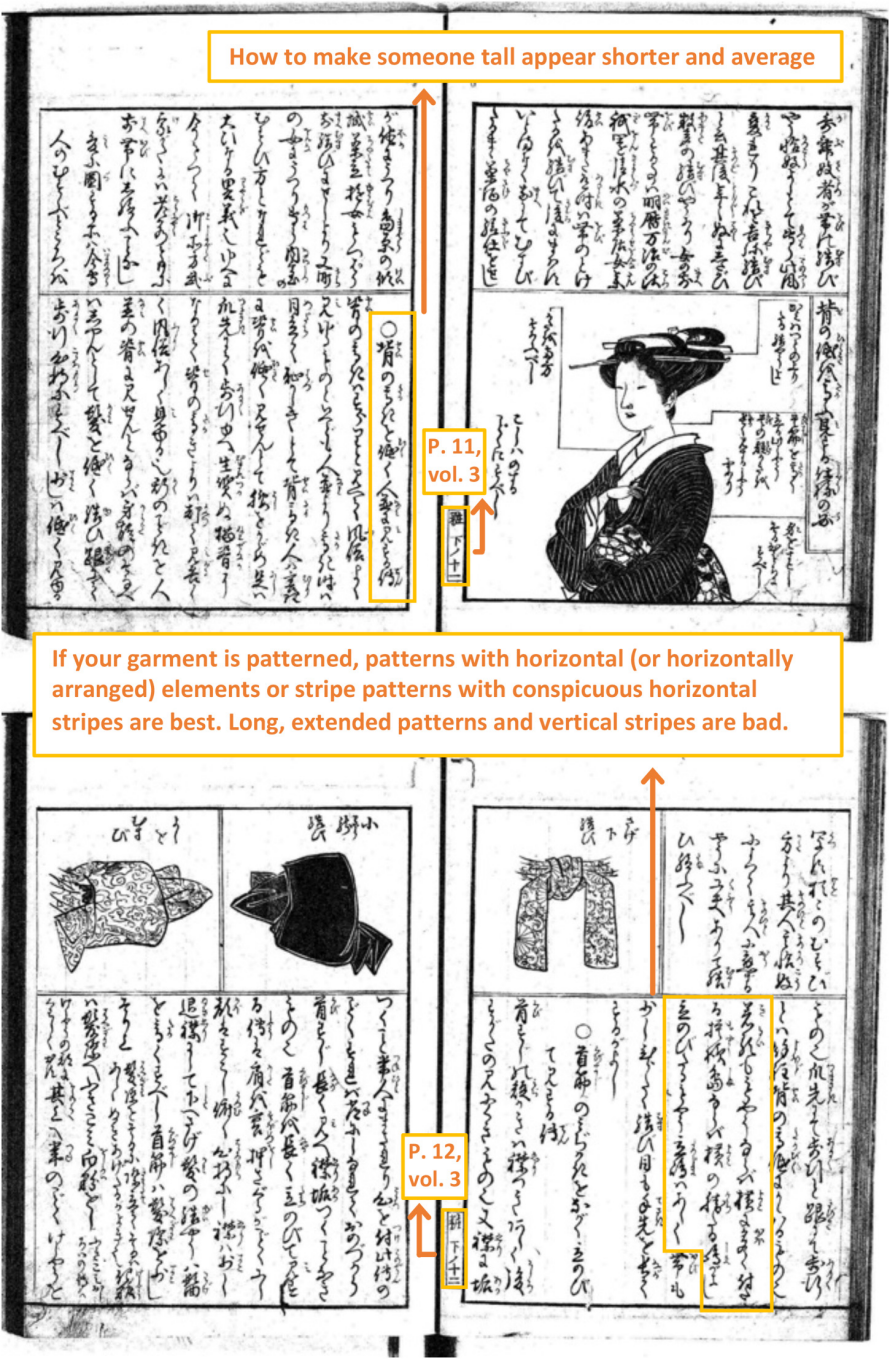
Pages 11–12, volume 3, “*Miyako Fuzoku Kewai Den*.”

*Note*. Created by modifying “*Miyako Fuzoku Kewai Den* (http://kotenseki.nijl.ac.jp/biblio/200005385).” The National Institute of Japanese Literature. Creative Commons Attribution License (CC BY-SA 4.0).

These pages contain sections describing how to look taller or shorter. Specifically, in the section titled “*Se no hikuki wo takaku misuru den* [How to make someone short appear taller] (Figure 1),” the following sentences are found: “Regarding tailoring, have your garments made slightly narrower for the body and choose long and tall flower prints. If the garment is striped, choose a vertical stripe or a pattern in which the vertical stripes are conspicuous. Do not choose patterns with conspicuous horizontal stripes or shapes.” Similarly, in the section titled “*Se no takaki wo hikuku hitonami ni misuru den* [How to make someone tall appear shorter and average] (Figure 2),” it is stated that horizontal stripes are effective in creating a shorter appearance and vertically striped patterns should be avoided. When read together, the common belief about stripes on clothes and body shape was already established in Japan in 1813.

In conclusion, the common belief was already established over 200 years ago in Japan, when vertical striped patterns on clothes became popular. This study is limited, as we only searched through Japanese literature from a specific period; it is unclear whether this belief existed in other cultures and historical times. The effect of striped clothes might also be different depending on the type or shape of the clothes. For example, the kimono (see the illustration at the top right of Figure 2), Japanese traditional wrapped-front clothes from shoulders to ankles, could enhance vertical straight lines and silhouette compared to clothes of standard length. Such vertical lines and silhouettes might be associated with the impression of a tall and slim body shape. Although these limitations remain, we found that the common belief about stripes on clothes and body shape was known, at least in the Far East, before Hermann von Helmholtz (1821–1894) was born, and it was applied in real life. Since stripes are a simple pattern and have long been used as prints on clothes in other cultures, the common belief that striped patterns on clothes modulate perceived body shape might have been observed in other cultures a long time ago. If so, why does the persistent and common belief conflict with the results of empirical studies (e.g., Thompson & Mikellidou, 2011) and the Helmholtz illusion (Helmholtz, 1867/1925, p. 193)? If the common belief is incorrect, how has the misbelief been shaped? The effect of striped clothes appears simple but is, in reality, complex.

## Supplemental Material

sj-pdf-1-ipe-10.1177_20416695221130779 - Supplemental material for Descriptions of a common belief in an 1813 Japanese beauty handbook regarding the influence of striped clothing on perceived body shape Click here for additional data file. Supplemental material, sj-pdf-1-ipe-10.1177_20416695221130779 for Descriptions of a common belief in an 1813 Japanese beauty handbook regarding the influence of striped clothing on perceived body shape by Yuki Miyazaki and Kentaro Ishibashi in i-Perception
