# Supplementary material for: Descriptions of a common belief in an 1813 Japanese beauty handbook regarding the influence of striped clothing on perceived body shape
Source: Iperception. 2022 Oct 12;13(5):20416695221130779. doi: 10.1177/20416695221130779 (PMC9558892; doi:10.1177/20416695221130779)
Supplement: sj-pdf-1-ipe-10.1177_20416695221130779 - Supplemental material for Descriptions of a common belief in an 1813 Japanese beauty handbook regarding the influence of striped clothing on perceived body shape [file sj-pdf-1-ipe-10.1177_20416695221130779.pdf]

Appendix

The following classical Japanese texts are direct quotations from Seyama and Hayami (1831/1982, pp. 224–227). The second author translated those into modern Japanese texts. The first author translated the texts into English. The yellow-highlighted parts are shown in the English translation version in Figures 1 and 2 of the paper.

○ 脊の低きを高く見する伝

生質の脊低きを高くせんは，その人の天然に違えば，なるべきことにあらず。人の脊，おおよそありて，至って高きと，至って低きといえども，二三寸にたがうことなし。されども高き人と低き人と並び歩行くに，至って目立ちて見ゆるものなりといえども，その違う所はわずかの寸ばかりなれば，身の動静にて脊をたかく見するしようあり。その伝は，脊すじを直に立て，肩を両方ひとしうそろえ，首を立ちのびるがごとくに押し上げ，身を少しそる心もちに腰をのすれば，すらりと恰好よく，高く見ゆる也。その上，下駄，草履のたぐいも，少し通例より高くして履くべし。髪も髷を高く結うがよし。衣類の仕立てようは，少し身巾のせまきかたに仕立てて，もようは，立ちのびたる草花の類を付くべし。嶋ならば立嶋か，立すじの勝ちたる嶋よし。横の勝ちたる嶋もようも，横にひらたき模様は悪しし。帯は少し細く仕立てて，結びようは手先をあまり長く出ださず，上の方にて高く結べば，およそは持ちあいにて人並みの高さに見ゆるもの也。
脊のひくきをかくさんとて，衣類を長く着て引きずり歩行き，自らは長く見せんと思えども，他よりは却って背の低きが目に付いて見ぐるしきものなれば，しゃんと着たるかたがよきなり。さて足どりは，指のさきを立ててあるく心もちなるがよし。背のひくき人の歩行風俗を見るに，おおく跟にてあるく心もちなるがゆえ，べたつきてなおさら背低う見ゆる也。なお図を見て知るべし。
すわりたる時は，帯を下にむすぶべし。常人の脊のごとく，少しもかわることなきなり。

○ 背の低きを高う見する仕様の図

- かみはつとの上がりたる結いようよし。
- 首筋を高く立てるけしょう首の短きを長く見するしようにあり。
- 身をすこしそる心もちにすべし。
- かたを両方そろうべし。
- 腰はのするごとくにすべし。

○ 背の高きを低く人並みに見する伝

脊の高きは，すらりと見えて風俗よく見ゆるものといえども，人並みより高き時は，目立ちて恥ずかしきとて，背高き人は害理に脊を低く見せんとて，腰をかがめ，足は爪先にて歩行くゆえ，生質ぬ猫脊になりて，脊の高きよりは，却って見苦しく風俗あしく見ゆる也。かくのごときを人並みの脊に見せんとならば，身体のそなえはしゃんとして，髪を低く結い，跟にて歩行く心持ちにすべし。少しは低く見ゆるもの也。爪先にて歩行くと，跟にて歩行くとは，余程背の高低にかかわるもの也。
着類ももようならば，横に多く付きたる模様，島ならば横の勝ちたる嶋がよし。立ちのびたるもよう，立嶋は悪しく，帯も少しひらたく，結び目も手先を長くするがよし。

Sayama, H., & Hayami, S. (1831/1982). *Miyako Fuzoku Kewai Den* [Cosmetic manners and customs in Edo] (in Japanese) (M. Tahakashi, Collation eds.), Tokyo: Heibonsya. (Original work published in 1813) (in Japanese). ISBN-13: 978-4582804140.
